# 

**Published:** 1917-12-08

**Authors:** 


### CONTENTS.

PAGE

**American and British Doctors and the G.M.C.** 195

**A War-Time Christmas in Hospitals** ... ... 196

**Hospital and Institutional News** ... ... ... 197

The Ministry of Health—Rations for Children—The Housing of the People—A Medical Officer's Action for Libel—The Moral of the Stepping Hill Case—The Fetish of Centralisation—A Word of Caution on Cornwall's Sanatorium—Women as Hospital Secretaries—Jubilee of the New Hospital for Women—Workmen's Contributions at Merthyr—Thinking in Decimal Points of a Penny—The New Medical Boards—Matches as a Source of Income—Cheap Milk for Babies—Form W. 3635 and V.A.D. Meals—The Craigleith Christmas Number—No More Castor Oil?—This Week's Drug Market—To Our Readers.

**"As Age Creeps On"** ... ... ... ... 201

Possible Troubles and How to Treat Them.

**Hay Camps** ... ... ... ... ... ... 202

The Economic Use of the Medical Profession.

**The Nation's Health** ... ... ... ... 203

Sir Arthur Newsholme's Annual Report, 1916-17.

**Treatment of Severe Facial Injuries** ... ... 205

By Major Gillies, Captain Kelsey Frey, and Mr. Percival Cole.

**New Appliances** ... ... ... ... ... 206

Hammock Suspension of Fractures.

PAGE

**Hospital and Institutional Results** ... ... 206

**The Editor's Letter-Box** ... ... 207

The Churches and Venereal Marriages: Do They Treat Marriage as a Sacrament or Not?

**The London Hospital** ... ... ... ... ... 207

Payment for Military Patients and Treatment of Discharged Warriors.

**Parliament and the Profession** ... ... ... 208

**The Matrons' and Sisters' Department** ... 209

A Study of Nursing Progress and its Development.

Round the Hospitals.

Are Liverpool's Nurses Awake?—The Strain on Homeland Matrons—The Loneliness of Their Lives—Every Matron a Sanctuary—An End to Sister Gorgons?—Homeland Workers and Battlefield Nurses.

**Midwifery in London** ... ... ... ... ... 213

**Forthcoming Meetings** ... ... ... ... 214

**Tragedies of War Bread** ... ... ... ... 214

**Gifts and Bequests** ... ... ... ... ... 214

**Matrons' and Sisters' Appointments** ... ... iv

**The Book Market** ... ... ... ... ... iv

**Passing Events and Topics** ... ... ... ... iv

**Institutional Needs** ... ... ... ... ... iv

**The Institutional Worker** ... ... (Suppl.) 1

Starting an Auxiliary Military Hospital.

